# Development of a Complex Intervention to Support the Use of Sedative Drugs in Specialist Palliative Care (iSedPall)

**DOI:** 10.1089/pmr.2024.0042

**Published:** 2024-11-29

**Authors:** Saskia Kauzner, Manuela Schneider, Maria Heckel, Carsten Klein, Claudia Bausewein, Eva Schildmann, Jeremias Bazata, Stefanie Kolmhuber, Sabine H. Krauss, Beatrice Odierna, Constanze Rémi, Jan Schildmann, Alexander Kremling, Christian Jäger, Kerstin Ziegler, Christoph Ostgathe

**Affiliations:** ^1^ Department of Palliative Medicine and Comprehensive Cancer Center, CCC Erlangen-EMN, University Hospital Erlangen, Friedrich-Alexander-Universität Erlangen-Nürnberg (FAU), Erlangen, Germany.; ^2^ Department of Palliative Medicine, LMU University Hospital Munich, Munich, Germany.; ^3^ Department for Palliative Medicine, Medical Faculty, University of Augsburg, Augsburg, Germany.; ^4^ Institute for History and Ethics of Medicine, Interdisciplinary Center for Health Sciences, Martin Luther University Halle-Wittenberg, Halle (Saale), Germany.; ^5^ Department of Criminal Law, Criminal Procedural Law, Commercial Criminal Law and Medical Criminal Law, Friedrich-Alexander-Universität Erlangen-Nürnberg (FAU), Erlangen, Germany.

**Keywords:** complex intervention, GUIDED, intervention development, palliative care, sedative drugs, theory of change

## Abstract

**Background:**

The option of intentional sedation to relieve intolerable suffering from treatment-refractory symptoms may elicit a feeling of safety for patients and informal caregivers as a last resort if the situation becomes unbearable. Many health care professionals feel uncomfortable and insecure in conducting intentional sedation due to specific challenges. We developed a complex intervention to support best practice use of sedative drugs in specialist palliative care in Germany based on previously published recommendations. This article aims at reporting the development of the intervention.

**Methods:**

The development of the intervention was based on theory and existing evidence with active stakeholder participation and patient and public involvement, following the updated Medical Research Council (MRC) Framework on complex interventions. A “Theory of Change,” drawing on expert-approved best practice recommendations and applying user-centered methods, fostered the development. The process encompassed study preparation, development of the elements of the intervention, and designing the multimodal intervention. For reporting, we adhere to the Guidance for Reporting Intervention Development framework.

**Results:**

The intervention is aimed at health care professionals working in specialist palliative care (inpatient and homecare settings) and consists of several components: (1) a screening tool, (2) the individual elements of the intervention, and (3) educational material for health care professionals to support them using the intervention. Additional information material was developed for patients and informal caregivers. Despite the benefits of stakeholder involvement, we faced some barriers due to limited health care staff and time resources and reservations regarding research in general.

**Discussion:**

A pilot study is planned for testing the overall feasibility of the intervention and exploring possible benefits for health care professionals to inform a subsequent fully powered implementation study. To deal with the challenges, we stayed in contact with the health care teams, maintained transparency, and provided opportunities for active participation.

## Key Message

We developed a complex intervention to support best practice of use of sedative drugs in specialist palliative care in inpatient and homecare settings based on previously developed recommendations. Stakeholders and a patient and public involvement group were integrated in the development process with the goal to design a feasible and suitable intervention for real-world settings.

## Background

When symptoms are refractory, a common and—at the same time—critically debated option in specialist palliative care is sedation.^1–4^ “Intentional sedation” is a new term in this context,^1^ commonly referred to as “palliative sedation”; in the clinical context of specialist palliative care, it refers to the use of sedative drugs with the intention of reducing the patient’s unbearable suffering due to treatment-refractory symptoms by reducing consciousness of variable duration (sedated temporarily or continuously until death) and depth (light or deep sedation).^5,6^ Offering intentional sedation as a last resort to patients and informal caregivers often seems to elicit a feeling of safety in case the situation becomes unbearable (e.g., for patients diagnosed with Amyotrophic lateral sclerosis) and of self-determination when they are actively involved in the decision-making process.^7,8^ At the same time, health care professionals sometimes experience moral distress due to a discrepancy in enabling the patient’s wish for ending the unbearable suffering by reduction of awareness and thus facilitating a “comfortable and calm” terminal stage of their illness and maintaining dignity.^7–12^ They also report unease in conducting intentional sedation due to specific medical (e.g., decision about an adequate dose of sedative drugs without shortening life), ethical (e.g., responding ethically to patients who wish to die), and legal challenges (e.g., decreased ability to communicate and for conscious decision making), which refer to the inpatient and homecare setting as well.^8,9,13–16^

However, for delivering patient-centered care in accordance with the patients’ values and wishes, especially at the end of life, it should be of utmost importance that health care professionals feel confident in their professional skills. To support best practice and medical decision making based on the current state of research, several guidelines and recommendations are provided nationally^17,18^ and internationally.^19^ For Germany, expert-approved best practice recommendations on sedative drug use and intentional sedation were developed by the SedPall study group based on the analysis of published guidelines on sedation in palliative care,^20–22^ disseminated nationally and internationally.^23,24^ The recommendations cover 10 topics (indications, intent/purpose, decision making, information and consent, medication and type of sedation, monitoring, management of fluids and nutrition, continuing other measures, support for relatives, and team support) on the whole spectrum of the use of sedative drugs from symptom control (restlessness and sleeplessness), sedation as a side effect, sedation used in terminating life-sustaining treatment, and emergency events (e.g., bleeding) to continuous deep sedation until death.^24^ In order to overcome the gap between the learning of professional skills and the navigation of these skills, it is necessary to provide hands-on supporting material for practical use in addition to available recommendations or guidelines.^25,26^

The iSedPall (“Development and piloting of a multimodal intervention for the recommended use of sedative drugs in specialist palliative care”) study group developed a complex intervention to support best practice of use of sedative drugs based on the previously developed recommendations (funded by the BMBF: 01GY2020A-C). The elements of this intervention aim to support medication-related decision making, patient information, documentation, and dealing with ethical challenges in inpatient and homecare settings in Germany (see Study Protocol, under review). The intervention will be piloted in four institutions of specialist palliative care (inpatient and homecare setting)–the so-called pilot centers–for 9 months.

This article aims at reporting the development of our complex intervention to provide transparency and for quality assurance since there is a perceived underreporting to date.^27^

## Methods

### Design

Due to the complexity of the intervention with multiple interacting components and different target groups, settings, and behaviors required by those who will use the intervention, we followed the updated MRC Framework for developing and evaluating complex interventions.^28,29^ A “Theory of Change” (ToC) was developed in advance to describe how and why the expected change should happen and to create a joint vision on the impact of the planned complex intervention.^30^ For putting the stakeholders in the focus of the developmental process, we integrated active stakeholder participation and patient and public involvement (PPI), thus considering different perspectives and needs (health care professionals, patients and/or informal caregivers, and the public). Additionally, we drew on theoretical expert guidance on how to develop complex interventions in health care settings.^30,31^ Due to the growing need for patient-centered and personalized care, health disciplines increasingly rely on user-centered design.^32–34^ The applied methods for gathering feedback from stakeholders, clinical experts, and PPI as informants, design partners, and testers during the developmental process were comparable with those methods used in the user-centered design process to guarantee a user-friendly result suitable for real-world settings.^35^ Describing the development process of the intervention is in adherence to the Guidance for Reporting Intervention Development (GUIDED)^27^ (see Supplementary Data S1). We also applied the Guidance for Reporting Involvement of Patients and the Public (GRIPP2—short form)^36^ (see Supplementary Data S2).

### Developmental process

The process of developing the complex intervention comprised three distinct work packages with occasional overlapping phases for (1) study preparation, (2) development of the individual elements of the intervention (supporting material for health care professionals), and (3) designing the complex intervention by assembling the single elements and preparing the implementation. Table 1 provides an overview of the work packages, which are also described below.

**Table 1.** Overview of Work Packages

Work package	1	2	3
Aim	Study preparation	Development of the elements of the intervention	Designing the multimodal intervention
Project month	1–6	4–13	13–18
Description	Developing theoretical basis Preparing for methodological work Exploring the evidence and user context	Consolidating the research results from work package 1 Defining the users’ needs and wishes Developing concept drafts of the elements of the intervention Gathering feedback and developing first prototypes of the elements of the intervention	Integration and final approval of the elements of the intervention Developing educational material Compiling the multimodal intervention and preparing the piloting phase
Methods	Literature reviews Theory of change workshops	Stakeholder interviews On-site visits Subproject-specific methods (e.g., group Delphi consensus procedure)	Workshops with stakeholders External expert feedback workshop Pretesting of the elements of the intervention with think-aloud interviews

All work packages were conducted in collaboration with patient and public involvement groups.

Four institutions formed the multidisciplinary research consortium with clinical (Palliative Medicine/Erlangen [consortium management] and Palliative Medicine/Munich), ethical (Medical Ethics/Halle), and legal (Medical Criminal Law/Erlangen) expertise. According to their respective expertise, for example, in the fields of medicine, gerontology, pharmacy, nursing science, psychology, sociology, ethics, and law, each institution formed one subproject covering the following topics: documentation, medication-related decision making, ethical challenging situations, and patient information. The elements of the intervention were developed within the subprojects with iterative feedback by all consortium members.

#### Work package 1: Study preparation

The first work package encompassed organizational, content, and method-related tasks as part of study preparation. For integration of participatory elements, a scientific advisory board and three PPI groups in Erlangen, Halle, and Munich were established. The German Association for Palliative Medicine delegated experts for the scientific advisory board.

The content-related and methodological work started with analyzing the previously developed best practice recommendations for the use of sedative drugs in palliative care.^23^ Furthermore, evidence was identified in reviews of literature that informed the development of the individual elements of the intervention.^37^

Then, to conceptualize the theoretical basis of the planned intervention, a ToC approach was applied. ToC does not refer to any preexisting theory but to the knowledge and expectations of the stakeholders.^38^ Three ToC workshops and several follow-up meetings were held in which representatives from the research consortium (*n* = 6), medical experts from the pilot centers who will apply the intervention during the pilot study (physicians: *n* = 1; nurses: *n* = 2), and representatives of the PPI groups (*n* = 2) participated. The participating stakeholders in this ToC process represented different perspectives (clinical, patients and informal caregivers, and the public).^39^ As the development of a ToC follows an iterative approach, it continued in work package 2.

#### Work package 2: Development of the elements of the intervention

After developing the theoretical and methodological framework of the intervention, the user context was examined more intensively to enrich the theoretical background with practical knowledge. Telephone interviews (*n* = 8) with staff from the pilot centers (head physicians and head nurses) were conducted to gain deeper insights into practical routines and challenges in the context of intentional sedation and to learn about their needs and expectations regarding the intervention from the perspective of the stakeholders.

In addition to remote interviews, on-site visits were scheduled at each pilot center to explore the user context. The users’ needs and wishes informed the first drafts of the elements of the intervention. Specific methods were applied for the development of some of the elements, e.g., a group Delphi consensus procedure (reported elsewhere). In the course of study preparation, we experienced the need for more guidance regarding situations for which the elements of the intervention would be appropriate. Following this, we developed a first draft of our screening tool for guiding the use of the intervention according to different patient scenarios. All elements were discussed during a consortium workshop including PPI members and adjusted subsequently. First prototypes of the elements of the intervention were prepared to be further tested in work package 3.

#### Work package 3: Designing the multimodal intervention

The prototypes were discussed in a workshop with relevant stakeholders and PPI members. Furthermore, a concept draft for educational material was presented and elaborated subsequently. The educational material is part of the intervention for supporting the implementation process at the pilot centers. After a revision phase with several feedback loops, external experts in the medical field from the scientific advisory board were invited to provide feedback on the revised elements during a workshop.

Before finalizing the intervention, the elements underwent a pretest^40^ based on case vignettes. Physicians and nurses (inpatient/home care setting; *n* = 12) were encouraged to run through the scenarios referring to intentional sedation and apply the elements of the intervention while thinking aloud. The material addressing patients, informal caregivers, or legal representatives was pretested with representatives of the local PPI groups. Subsequently, the multimodal intervention was finalized and consented in the study group leading to the preparation of the following prepiloting phase.

### Prepiloting phase

Commencing in February 2023, the intervention was applied in four pilot centers, two inpatient specialist palliative care units, and two specialist palliative homecare teams, for 3 months in the sense of prepiloting. The health care teams were encouraged to use the intervention when dealing with potentially sedative drugs. Then, case-based feedback sessions were held with health care professionals to gain insight into first experiences in using the elements of the intervention and possible barriers to usage. Subsequently, necessary adaptations of the elements were deduced and integrated accordingly to prepare the actual pilot phase.

### Patient and public involvement

The local PPI groups consisted of representatives of the public, patients, and informal caregivers who had personal experience with palliative care or sedation. In most cases, PPI members had lost a close family member or friend due to terminal illness, with some of them being sedated at the end of life. Several members already took part in the previous project where the recommendations were developed; others were recruited by consortium members through personal contact or by members of the PPI groups themselves. We appreciated to have a perspective of the public to learn how to reach out to society for disseminating our study results as well as a perspective on how to raise awareness for palliative care in general. We also hoped that patients and informal caregivers help us in understanding the nonprofessional perspective when caring for a sedated relative. After networking meetings and introductory educational sessions on relevant research methods for the PPI groups, participatory elements during the developmental process were jointly identified in work package 1. The PPI members were actively participating in the ToC process, in the workshops with stakeholders and external experts and provided input and feedback through the pretests regarding the material directly addressing patients and/or informal caregivers (e.g., related to informed consent). The contribution of the PPI groups was documented during the development process and reported back to the groups after the elements of the intervention were finalized.

## Results

The developmental phase lasted 18 months, commencing in August 2021. Ten meetings and six workshops were scheduled within the research consortium with the support of relevant stakeholders, medical experts, and PPI members to develop our intervention. The following piloting phase (start: May 2023) for testing the feasibility of the intervention will be described elsewhere.

### Developmental activities

**FIG. 1.**
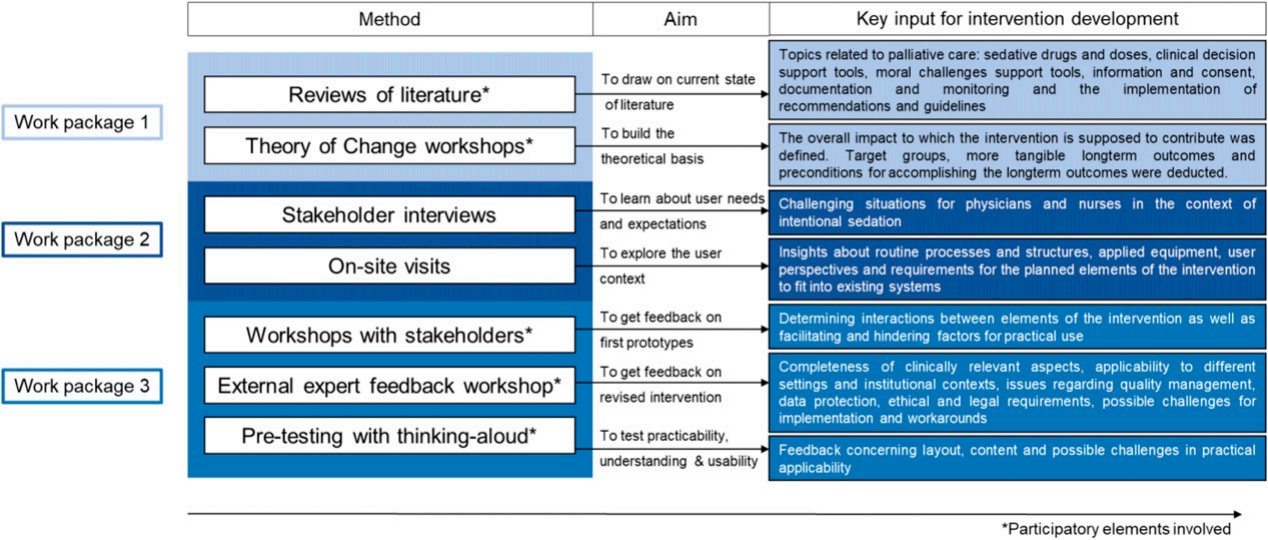
Evidence from different sources: Overview of applied methods and key input for intervention development.

Figure 1 provides an overview of the different sources of evidence that informed the developmental process. The key input will be reported in the following.

#### Work package 1

After scoping literature on methodology, we decided on the MRC Framework for developing and evaluating complex interventions as evidence base for the development of our complex intervention. By analyzing the best practice recommendations for using sedative drugs, we established a joint definition of intentional sedation to relieve intolerable suffering within the research consortium, the stakeholders, and PPI groups, and we also revealed the most relevant topics to be addressed by the intervention: medication, information and consent, documentation, and moral challenge analysis.

Reviews of literature depicted the current state of literature regarding the following topics: sedative drugs and doses, clinical decision support tools, moral challenges support tools, information and consent, documentation and monitoring, and the implementation of recommendations and guidelines.

The first draft of the ToC informed the development of the elements of the intervention mainly by identifying different target groups (patients, informal caregivers, and health care professionals) to ensure that the respective needs and expectations regarding the intervention were taken into account. Determining the overall impact (patient-centered care) and long-term outcomes with specific preconditions (e.g., feeling confident in professional skills) helped to shape first drafts of the elements and to anticipate potential barriers in usage, such as differing staff capacities or lack of equipment.

#### Work package 2

Stakeholder interviews revealed the following issues as being challenging for physicians: administering the “right” dose for adequate symptom relief and predicting possible drug reactions, a sometimes fluent transition from intermittent sedation to sedation until death with a change in intention (symptom treatment in acute situations vs. intentionally reducing consciousness), decision making when the patient is not able to give consent, and the documentation of all relevant issues. Interviewed nurses described the following as challenging: the fear of not being able to deal with the situation during the night when physicians are not immediately available, working with analog and digital documentation forms at the same time (prone to error), enormous personal effort before (decision making and involvement of a complete caring network), and during sedation (close-meshed monitoring and support of informal caregivers) especially in the home care setting, and dealing with family conflicts, which might be ethically challenging.

By attending team meetings and patient visits during on-site visits at the pilot centers, interactions, routine processes, structures, and equipment (e.g., digital documentation systems) were examined. Staff provided insight into user perspectives and requirements for the planned intervention. This was especially insightful in terms of setting-specific differences in practice, for example, the involvement of informal caregivers in the homecare setting.

These reported challenges and requirements were addressed in our intervention by developing supporting material to be used in the multidisciplinary team and for both settings. We concluded that there is a need for our intervention. At the same time, the health care teams already seemed to be confident and experienced in their daily practice, so we tried to enhance their commitment by offering several possibilities for codesigning the elements of our intervention in work package 3.

#### Work package 3

By discussing the prototypes within a workshop with relevant stakeholders and PPI members, we got feedback regarding overlap and possible interactions between the elements and potentially facilitating and hindering factors for practical use. Therefore, it seemed relevant to reduce the scope of the material and prioritize some elements of the intervention, for example, for acute situations. Feedback regarding the concept for the educational material revealed individual needs and preferences of the different health care teams, for example, individual versus team training and online versus on-site training. Within a following workshop with external experts in the medical field, completeness of clinically relevant aspects, applicability to different settings and institutional contexts, issues regarding quality management, data protection, ethical and legal requirements, possible challenges for implementation, and workarounds were discussed.

Pretesting of the elements of the intervention with physicians and nurses focused on practicability, understanding, and usability. For the documentation template, we noticed uncertainties referring to wording and layout, some missing data, and got suggestions for removing items.

The elements of the intervention addressed to patients and informal caregivers (e.g., handout for informal caregivers of sedated patients and information sheet for patients) were pretested with PPI. Their feedback helped us shape the elements of our intervention in terms of understandability, usability, and acceptability. Furthermore, the involvement of the nonprofessional perspectives of the PPI members revealed possible gaps between theory and practice and how to address them.

Involving stakeholders and PPI in developing the intervention was very valuable, but challenges arose. Emerging obstacles referred to the limited availability of health care professionals due to staff shortages and limited time resources in clinical practice and reservations with respect to research in general.

### Intervention

The final intervention comprises (1) a screening tool for guiding the use of the intervention, (2) the individual elements of the intervention for the use of sedative drugs, and (3) educational material for health care professionals to support them using the intervention. The elements of the intervention are meant for health care professionals in specialist palliative care inpatient and homecare settings. Additional information material was delivered for patients and informal caregivers on demand. Table 2 gives an overview of the multimodal intervention. Furthermore, exemplary best practice recommendations as the basis for the elements’ development are outlined.^24^ The detailed overview of the elements and description of the intervention and its user context are presented in the Study Protocol (under review) according to TiDieR checklist.^41^

**Table 2.** Overview of the Multimodal Intervention

Elements	Content	Best practice recommendation (topic, number)
**Screening tool**: “When should the elements of the intervention be used?”	Guiding the application of the elements of the intervention in different situations: (1) sedative drug effects anticipated, (2) intentional sedation planned, and 3) reduced consciousness possibly medication-induced	
Elements of the intervention	Providing support for medical, ethical, and legal considerations in relation to intentional sedation	
Medication	Warning list to support the clinical judgement if a certain dose of a potentially sedative drug is to be expected to have sedative effects on the patient Expert-based recommendations regarding sedative drug doses for initiating intentional sedation	Decision making, 7 Medication and types of sedation, 2
Information and consent	Information sheets for patients and legal representatives regarding intentional sedation in detail Checklist on information provision for physicians providing an overview of the most legally relevant topics Handout for informal caregivers to prepare for supporting a sedated patient	Information and consent, 6 Information and consent, 1 Support for relatives, 4
Documentation	Documentation templates for health professionals in specialist palliative care and informal caregivers in the homecare setting with all relevant aspects before (planning) and during (monitoring) an intentional sedation	Decision making, 8
Moral challenge analysis	Ethical screening tool to guide the use of the ethical material Analyses of six ethically challenging situations from the perspective of medical ethics Checklists for deliberation referring to each analysed ethically challenging situation guiding ethical case discussions or team meetings Information brochure for patients and informal caregivers to prevent the ethically challenging situations by providing additional information on intentional sedation	Indication, 7
Supplementary material	Educational short videoclips for health care professionals to guide the application of the elements of the intervention and support the implementation	

### Prepiloting phase

The adjustments conducted after the prepiloting phase based on the feedback of the pilot centers, mainly encompassed the content of the elements of the intervention: clarifications and supplements to the content, change in the order of usage, adjustments of the layout and wording, and supplementation of a figure to guide the usage of the ethical material. The adaptations made had no significant effect on the intended application of the elements and were not setting-specific. The final elements of the intervention were then provided to all pilot centers for a pilot phase of 9 months. The results of the pilot study will be published elsewhere.

## Discussion

The multimodal intervention was developed on the basis of theory, evidence with active involvement of stakeholders, and PPI. The intervention is currently piloted for 9 months to test its overall feasibility and its possible benefit for health care professionals when using sedative drugs in specialist palliative care. By providing assistance for these sedation-specific tasks, we intend to strengthen the professional skills of health care providers and thereby support the best practice of use of sedative drugs in specialist palliative care. By doing so, we are—to the best of our knowledge—the first to provide hands-on supporting material for clinical practice based on best practice recommendations helping to bridge the gap between learning and navigating professional skills. Beyond that, we thrive to raise awareness for potential sedative drug effects to ensure patient-centered care.

Despite thorough exploration of potential adaptations of the intervention during the developmental phase, it is challenging to fully anticipate differences in the utilization of the elements of the intervention depending on the setting or characteristics of the institution itself. Such variations with necessary context-specific adjustments will only become apparent following the piloting phase. That is why it is essential to combine a more theoretical developmental phase with an evaluation (piloting) phase before starting to implement the intervention in practice—in line with the MRC Framework for developing complex interventions.

Developing a complex intervention with multimodal elements can be challenging since you have to continuously check on the fit of the single elements with each other from the very beginning and you need to anticipate the practical application of the elements. In our case, the documentation template and the checklist on information provision had some overlapping information, which had to be aligned. For this reason, it is essential to regularly stay in touch within the research team and to early integrate relevant stakeholders for gathering external feedback. Furthermore, you need to have the interaction of the multimodal elements in mind for finally setting up the intervention. For this reason, we developed a screening tool for guiding the use of the intervention, which was subsequently added to the project plan. The development of a ToC as an iterative process is very time-consuming and exceeded the planned schedule. Therefore, we recommend to schedule enough resources in the project plan, especially for the main responsible person in this process.

In line with user-centered design, we focused on the stakeholders’ needs and applied a spectrum of methods to explore the user context and perspective in-depth (interviews, on-site visits, stakeholder workshops, and ToC approach) as the basis for our developmental process. Due to the sensitivity and the existential nature of the topic “sedation in palliative care” for both the health care professionals and the patients and their informal caregivers, it was of utmost importance to us to involve those stakeholders from the very beginning to develop an intervention that is really needed and helpful in practice and that supports patient-centered care. We empowered the clinical stakeholders and the local PPI groups as informants, testers, and design partners^35^ by jointly developing the elements of the intervention and by providing the opportunity to give feedback and revise elements of the intervention.

Despite the valuable input through stakeholder involvement and PPI, we came across barriers. To address these issues, we stayed in constant contact with the stakeholders, maintained transparency in the development process, emphasized the value of collaboration between research and practice, and provided opportunities for active participation.

### Strengths and limitations

Following standardized guidelines for reporting key aspects of intervention development serves as quality assurance, provides transparency for researchers, funders, and the public, and supports the selection of an adequate development approach for effective interventions.^27^ To date, intervention development processes are underreported, and reporting guidance does barely exist.^27,41,42^ For this reason, it is a strength of this article to adhere to the GUIDED framework. The intervention development within an interdisciplinary research consortium safeguarded the integration of the medical, ethical, and legal perspective and helped to consider empirical knowledge and practical experience at the same time.

The elements of the intervention are currently available in German only and focus on the German culture and legal system, which may be a limiting factor. Furthermore, due to the user-centered design, the elements of the intervention were developed according to the specific needs and expectations of our four pilot centers. To overcome this potential bias, we additionally drew on evidence-based literature, for example, by a systematically conducted Scoping Review. A possible subsequent implementation study should take these limitations into account by providing multilingual elements of the intervention and by testing the generalizability of the intervention to other palliative care services where intentional sedation to relieve intolerable suffering is initiated. By providing educational material in the form of self-training video clips for supporting the use of the elements of the intervention, we achieved our project milestone. Nevertheless, an implementation study should prioritize this topic by involving professionals in education with dedicated competencies.

## Conclusions

For developing our complex intervention, we applied a theory-based, user-centered developmental approach by drawing on expert-based guidance on developing complex interventions with integration of stakeholders and PPI in different stages and scopes, reported by using the GRIPP2—short form. The elements of the intervention, referring to medication, information and consent, documentation, and moral challenge analysis, are meant to be practical and meaningful to health care professionals in real-world settings with the aim of supporting the use of sedative drugs in specialist palliative care, which will be explored during the pilot phase. For transparency, quality assurance, and comparability, we adhere to the GUIDED framework for reporting the development process of the intervention. The findings of the following pilot study regarding overall feasibility will inform a subsequent implementation study.

## Abbreviations Used

ALS: Amyotrophic lateral sclerosis
MRC: Medical Research Council
