# Supplementary material for: Development of a Complex Intervention to Support the Use of Sedative Drugs in Specialist Palliative Care (iSedPall)
Source: Palliat Med Rep. 2024 Nov 29;5(1):527–36. doi: 10.1089/pmr.2024.0042 (PMC11864855; doi:10.1089/pmr.2024.0042)
Supplement: Supplementary Data S1 [file pmr.2024.0042_supp_datas1.docx]

Supplementary File 1:

**GUIDED – a guideline for reporting for intervention development studies**

Duncan E, et al. BMJ Open 2020; 10:e033516. doi: 10.1136/bmjopen-2019-033516

| **Item description** | **Page in manuscript where item is located** | **Other**  e.g. if item is reported elsewhere, then the location of this information can be stated here. |
| --- | --- | --- |
| 1. Report the **context** for which the intervention was developed. | 2 |  |
| 1. Report the **purpose** of the intervention development process. | 3 |  |
| 1. Report the **target population** for the intervention development process. | 8 |  |
| 1. Report how any **published intervention development approach** contributed to the development process. | 3, 6 |  |
| 1. Report how **evidence from different sources** informed the intervention development process. | 6-8  (fig. 1) |  |
| 1. Report how/if **published theory** informed the intervention development process. | 3-4 |  |
| 1. Report any **use of components from an existing intervention** in the current intervention development process. | n.a. |  |
| 1. Report any **guiding principles, people or factors** that were prioritised when making decisions during the intervention development process. | 3-4 |  |
| 1. Report how **stakeholders** contributed to the intervention development process. | 3-4, 5-7 |  |
| 1. Report how the intervention **changed** in **content** and **format** from the start of the intervention development process. | 8 |  |
| 1. Report any **changes** to intervention required or likely to be required for **subgroups.** | 8 |  |
| 1. Report important **uncertainties** at the end of the intervention development process. | 9 |  |
| 1. Follow **TIDieR guidance** when describing the developed intervention. | 8 | See Study Protocol |
| 1. Report the intervention development process in an **open access format.** |  | Fulfilled |

*Note*: n.a. = not applicable.
