# Supplementary material for: Development of a Complex Intervention to Support the Use of Sedative Drugs in Specialist Palliative Care (iSedPall)
Source: Palliat Med Rep. 2024 Nov 29;5(1):527–36. doi: 10.1089/pmr.2024.0042 (PMC11864855; doi:10.1089/pmr.2024.0042)
Supplement: Supplementary Data S2 [file pmr.2024.0042_supp_datas2.docx]

Supplementary File 2:

**GRIPP2 reporting checklist (short form) – reporting of patient and public involvement in research**

Staniszewska S, et al. BMJ Open 2017; 358:j3453. doi: 10.1136/bmj.j3453

| **Section and topic** | **Item** | **Reported on page No** |
| --- | --- | --- |
| 1: Aim 3 | Report the aim of PPI in the study | 5 |
| 2: Methods | Provide a clear description of the methods used for PPI in the study | 5-6 |
| 3: Study results | Outcomes—Report the results of PPI in the study, including both  positive and negative outcomes | 6-8 |
| 4: Discussion and conclusions | Outcomes—Comment on the extent to which PPI influenced the study  overall. Describe positive and negative effects | 9 |
| 5: Reflections/critical perspectives | Comment critically on the study, reflecting on the things that went well and those that did not, so others can learn from this experience | 9 |

*Note*: PPI = patient and public involvement.
